# KKL-35 Exhibits Potent Antibiotic Activity against Legionella Species Independently of *trans*-Translation Inhibition

**DOI:** 10.1128/AAC.01459-17

**Published:** 2018-01-25

**Authors:** Romain Brunel, Ghislaine Descours, Isabelle Durieux, Patricia Doublet, Sophie Jarraud, Xavier Charpentier

**Affiliations:** a CIRI, Centre International de Recherche en Infectiologie, Team Horizontal Gene Transfer in Bacterial Pathogens, INSERM, U1111, Université Claude Bernard Lyon 1, CNRS, UMR5308, École Normale Supérieure de Lyon, Université Lyon, Villeurbanne, France; b CIRI, Centre International de Recherche en Infectiologie, Team Pathogenesis of Legionella, INSERM, U1111, Université Claude Bernard Lyon 1, CNRS, UMR5308, École Normale Supérieure de Lyon, Université Lyon, Villeurbanne, France; c Centre National de Référence des Légionelles, Centre de Biologie et de Pathologie Est, Bron, France

**Keywords:** Legionella, *trans*-translation

## Abstract

*trans*-Translation is a ribosome-rescue system that is ubiquitous in bacteria. Small molecules defining a new family of oxadiazole compounds that inhibit *trans*-translation have been found to have broad-spectrum antibiotic activity. We sought to determine the activity of KKL-35, a potent member of the oxadiazole family, against the human pathogen Legionella pneumophila and other related species that can also cause Legionnaires' disease (LD). Consistent with the essential nature of *trans*-translation in L. pneumophila, KKL-35 inhibited the growth of all tested strains at submicromolar concentrations. KKL-35 was also active against other LD-causing Legionella species. KKL-35 remained equally active against L. pneumophila mutants that have evolved resistance to macrolides. KKL-35 inhibited the multiplication of L. pneumophila in human macrophages at several stages of infection. No resistant mutants could be obtained, even during extended and chronic exposure. Surprisingly, KKL-35 was not synergistic with other ribosome-targeting antibiotics and did not induce the filamentation phenotype observed in cells defective for *trans*-translation. Importantly, KKL-35 remained active against L. pneumophila mutants expressing an alternate ribosome-rescue system and lacking transfer-messenger RNA, the essential component of *trans*-translation. These results indicate that the antibiotic activity of KKL-35 is not related to the specific inhibition of *trans*-translation and its mode of action remains to be identified. In conclusion, KKL-35 is an effective antibacterial agent against the intracellular pathogen L. pneumophila with no detectable resistance development. However, further studies are needed to better understand its mechanism of action and to assess further the potential of oxadiazoles in treatment.

## INTRODUCTION

Legionella pneumophila is a ubiquitous freshwater bacterium that infects a wide spectrum of environmental protozoans. Human-made systems, such as sanitary water networks and air-cooling towers, can disseminate contaminated water through aerosolization. The breathing of microscopic droplets contaminated with L. pneumophila can lead to infection of alveolar macrophages and development of a life-threatening pneumonia called Legionnaires' disease (LD) or legionellosis. LD remains an important cause of both morbidity and mortality in Europe, with over 6,900 cases being reported in 2014 (1). Guidelines for the management of LD recommend the use of macrolides (with a preference for azithromycin) or fluoroquinolones (levofloxacin or moxifloxacin) to treat the infection (2, 3). Despite a rapid diagnosis and the correct administration of antibiotics, the death rate among those with LD is over 10% (4). L. pneumophila isolates are considered susceptible to macrolides and fluoroquinolones (5), but mutants resistant to both antibiotic families can easily be obtained *in vitro*, suggesting that resistant strains may emerge during treatment (6–8). Indeed, the acquisition of resistance to fluoroquinolones during the course of fluoroquinolone therapy has recently been reported (9, 10). New compounds that are active against L. pneumophila strains resistant to fluoroquinolones and macrolides or that could potentiate these existing treatments may improve the outcome of the disease.

*trans*-Translation has recently been proposed to be a novel target for the development of a new class of antibiotics (11). *trans*-Translation is the primary bacterial mechanism used to resolve ribosome stalling in bacteria (12–14). Ribosome stalling can be induced by translation of an mRNA lacking a stop codon (non-stop mRNA) or when ribosomes pause before the stop codon is read (i.e., due to ribosome-targeting antibiotics, rare sense codon stretches, a lack of necessary tRNAs, etc). Ribosome stalling is a life-threatening issue in metabolically active bacteria (15, 16). *trans*-Translation is operated by a highly conserved nucleoprotein complex (17) encoded by two genes: *ssrA*, encoding a highly expressed and structured RNA called transfer-messenger RNA (tmRNA) (18, 19), and *smpB*, encoding a small protein involved in the specific recognition and loading of tmRNA in stalled ribosomes (20–22). Once the complex is loaded into the free A site of the stalled ribosome, translation resumes using the coding section of the tmRNA as the template. This messenger section of tmRNA encodes a degradation tag that is appended to the unfinished polypeptide, targeting it to different proteases (23–25). The coding section of tmRNA ends with a stop codon, allowing the normal termination of translation and dissociation of the ribosomal subunits. In addition, the tmRNA-SmpB complex interacts with RNase R to degrade the faulty mRNA (26, 27). Thus, in addition to resolving ribosome stalling, the *trans*-translation system prevents the rise of further problems by promoting the degradation of both the problematic mRNA and the aborted polypeptide (28).

Alternative ribosome-rescue systems have been identified in Escherichia coli and named ArfA and ArfB (alternative rescue factors A and B, respectively) (29–31). Both ArfA and ArfB can partially complement the loss of *trans*-translation by promoting the dissociation of the stalled ribosome but lack the mechanisms to trigger degradation of the aborted polypeptide and faulty mRNA (12). These appear to be less conserved than the tmRNA-SmpB system (15). *trans*-Translation is essential in species lacking alternative mechanisms (16). In agreement with these observations, alternative ribosome-rescue systems are absent in members of the Legionellaceae family, and we indeed found that *trans*-translation is essential for L. pneumophila growth and infection of its cellular host (32). In L. pneumophila, expression of the alternate rescue factor ArfA from E. coli can compensate for the loss of *trans*-translation activity, indicating that the ribosome-dissociating activity of the *trans*-translation system is the sole function required for viability (32). Because it is essential for viability in multiple pathogens, the *trans*-translation system has been proposed to be a valid yet unexplored target for a new class of antibiotics (11).

A high-throughput screen using an *in vivo* assay of *trans*-translation recently identified a family of small molecules able to inhibit *trans*-translation at micromolar concentrations (33). One of the most active compounds, KKL-35, was found to exhibit bactericidal activity against several pathogenic bacterial species in which *trans*-translation was known to be essential (33). KKL-35 and two related compounds, KKL-10 and KKL-40, displayed antibiotic activity against the intracellular pathogen Francisella tularensis during infection of its host (34). However, the specificity of action of the molecules has not been confirmed in this species. The present study assessed the activity of KKL-35 against the intracellular pathogen L. pneumophila. We report that KKL-35 exhibits potent antibiotic activity against L. pneumophila at very low concentrations and is able to stop bacterial multiplication in a model of infection of human macrophages, yet multiple pieces of evidence indicate that KKL-35 does not target *trans*-translation, and as such, its true target(s) in L. pneumophila remains to be identified.

## RESULTS

### KKL-35 inhibits Legionella growth *in vitro*.

**FIG 1:**
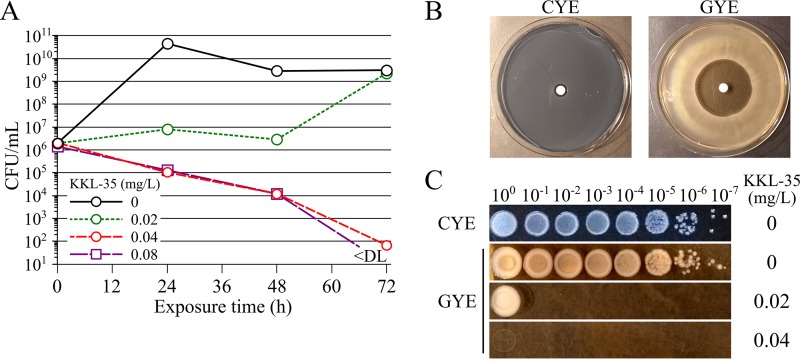
Antibiotic activity of KKL-35 against L. pneumophila in liquid and solid media. (A) Time-kill analysis of the activity of KKL-35 against L. pneumophila in AYE liquid medium. L. pneumophila strain Paris was resuspended in AYE medium at 3 × 10^6^ CFU/ml with a range of 2-fold dilutions of KKL-35. The tubes were then incubated at 37°C. Every 24 h, serial dilutions were plated on CYE agar and the numbers of CFU were counted. The data presented are averages for triplicate samples. The data presented are representative of those from three experiments performed independently. (B) Antibiotic activity of KKL-35 in a solid medium disk diffusion assay. A paper disk containing 100 μg of KKL-35 was placed at the center of a CYE or GYE plate, which was inoculated by flooding with a suspension of L. pneumophila. (C) Determination of the MIC of KKL-35 on GYE plates. Serial 10-fold dilutions of a culture of L. pneumophila in stationary phase (∼5 × 10^9^ CFU/ml) were spotted (10 μl) on CYE (no KKL-35) and GYE plates containing increasing concentrations of KKL-35.

The MICs of KKL-35 for five L. pneumophila strains and three non-L. pneumophila species causing LD were determined *in vitro* using the broth microdilution method (Table 1). KKL-35 strongly inhibited the growth of all tested species and was particularly potent against the species Legionella pneumophila, with all tested strains exhibiting a MIC of about 0.04 mg/liter. A time-kill assay with L. pneumophila strain Paris showed the bactericidal activity of KKL-35, with a decrease in viability being seen at 24 h after addition of KKL-35 at concentrations equal to or higher than the MIC (Fig. 1A). At 72 h following addition of KKL-35 at the MIC, the viable count was reduced by 4 orders of magnitude. Exposure to KKL-35 at half the MIC led to transient bacteriostatic activity for 48 h, but then this was followed by growth, suggesting that KKL-35 degrades and loses activity under those conditions. We also tested the activity of KKL-35 against 12 L. pneumophila mutants that were evolved from the Paris strain to become highly resistant to erythromycin and azithromycin (4,000-fold increases in the MICs) (8). The MIC of KKL-35 for these mutants was identical to that for the parent strain (0.04 mg/liter) and was thus unaffected by ribosomal mutations involved in macrolide resistance (23S rRNA and L4 and L22 protein mutations). Interestingly, KKL-35 was poorly active in conventional charcoal-yeast extract (CYE) solid medium for L. pneumophila isolates with MICs of >10 mg/ml. A paper disk containing 100 μg of KKL-35 produced an inhibition zone of 7 to 8 mm in diameter (Fig. 1B). To test the possibility that the agar-charcoal gelling base of CYE plates reduced the activity of KKL-35, we replaced it with the guar gum gelling agent. On guar gum-yeast extract (GYE) plates, L. pneumophila formed colonies exactly like those on CYE plates (Fig. 1C), but a disk of 100 μg of KKL-35 produced an inhibition zone of 40 mm in diameter (Fig. 1B). KKL-35 at 0.02 g/liter could already inhibit the growth of the inoculum with less than 10^7^ CFU, and at 0.04 g/liter (the MIC in broth), no growth could be observed even with the highest inoculum (∼10^8^ CFU) (Fig. 1C). Thus, KKL-35 at a low concentration inhibited L. pneumophila growth both in liquid medium and in solid medium.

**TABLE 1:** MICs of KKL-35 for several Legionella species *in vitro*

Strain	MIC^*a*^ (mg/liter)
L. pneumophila Paris	0.04 ± 0
L. pneumophila Lens	0.04 ± 0
L. pneumophila Lorraine	0.04 ± 0
L. pneumophila Philadelphia-1	0.067 ± 0.062
L. pneumophila 130b	0.04 ± 0
L. longbeachae ATCC 33484	0.08 ± 0
L. micdadei ATCC 33218	0.08 ± 0
L. dumoffii ATCC 35280	0.08 ± 0

a The values are the averages ± standard deviations from three independent determinations.

### KKL-35 inhibits intracellular growth of L. pneumophila.

**FIG 2:**
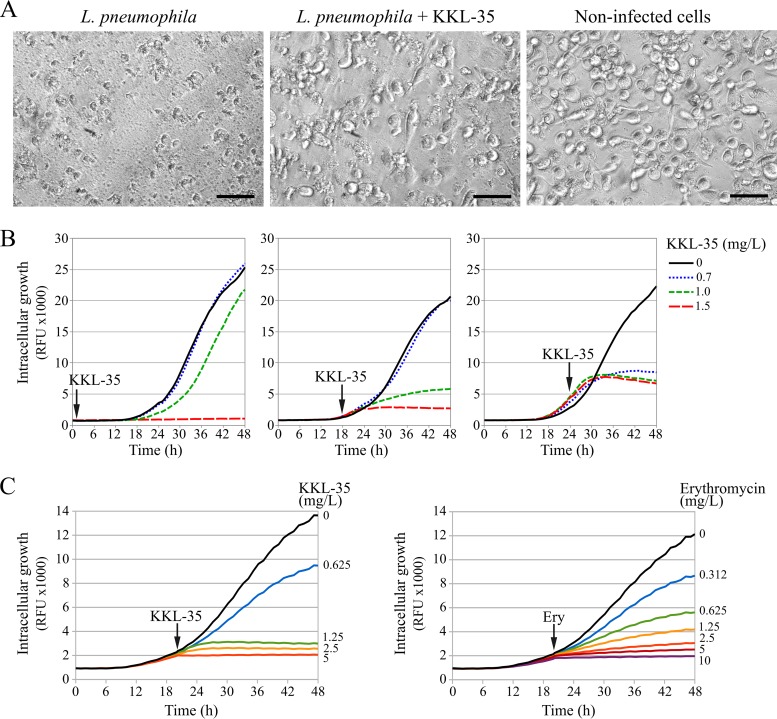
Activity of KKL-35 against L. pneumophila in an intracellular replication model. (A) Bright-field microscopy imaging of U937-derived macrophages infected with L. pneumophila (MOI = 10) for 72 h in the presence of absence of KKL-35 at 10 mg/liter. Bars, 50 μm. (B) Live monitoring of intracellular replication of GFP-producing L. pneumophila strain Paris carrying plasmid pX5 in U937-derived macrophages. KKL-35 was added at 1 h, 18 h, or 24 h postinfection. GFP fluorescence levels were automatically monitored every hour for 48 h. RFU, relative fluorescence units. Data are averages for three wells and are representative of those from an experiment performed three times independently. (C) Comparison of the activity of KKL-35 and erythromycin on the intracellular replication of L. pneumophila. KKL-35 and erythromycin were added at 20 h postinfection. Data are averages for three wells and are representative of those from an experiment performed twice independently.

L. pneumophila can infect human macrophages and replicate extensively within a membrane-bound compartment until cell lysis. Two molecules, KKL-10 and KKL-40, structurally related to KKL-35 were found to be nontoxic to macrophages at concentrations up to 19 mg/liter (34). Indeed, we found that KKL-35 was not toxic at 10 mg/liter and even protected monocyte-derived macrophages from killing by L. pneumophila at a multiplicity of infection (MOI) of 10 (Fig. 2A). In order to better characterize the inhibitory activity of KKL-35, we used a green fluorescent protein (GFP)-based time-resolved assay to follow the replication of GFP-expressing L. pneumophila in monocyte-derived macrophages (35). Within minutes of forced contact with macrophages, L. pneumophila is internalized in a vacuolar compartment that escapes fusion with lysosomes (36, 37). Addition of KKL-35 at 1 h after infection, when bacteria are intracellular but not yet multiplying, prevented L. pneumophila replication at concentrations above 1 mg/liter (Fig. 2B). Moreover, when added at later time points (18 or 24 h), when multiplication is ongoing, KKL-35 could inhibit replication at even lower concentrations (0.7 mg/liter) (Fig. 2B). This may indicate either that KKL-35 is more active against actively dividing cells or that the active fraction of KKL-35 gradually decreases over time. The ability of KKL-35 to completely halt replication was then compared to the activity of the macrolide erythromycin, a recommended treatment for LD. When added to actively replicating L. pneumophila, erythromycin began to inhibit replication at 0.31 mg/liter (Fig. 2C). Each 2-fold increase in concentration further inhibited replication. A nearly complete and immediate inhibition of replication was obtained at a concentration 32 times higher than the first inhibitory concentration (10 mg/liter). While KKL-35 began to inhibit replication at a concentration of 0.62 mg/liter, it completely stopped replication at a concentration only 8 times higher (5 mg/liter) (Fig. 2C). This indicates that KKL-35 may be more bactericidal than erythromycin. Altogether, the data show that KKL-35 inhibits the replication of L. pneumophila within macrophages.

### KKL-35 does not induce phenotypes associated with a loss of *trans*-translation.

**FIG 3:**
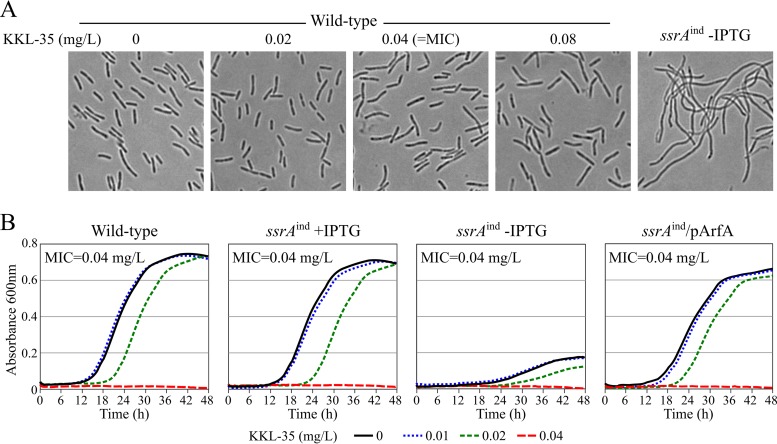
KKL-35 does not primarily target *trans*-translation in L. pneumophila. (A) Phase-contrast light microscopy of wild-type L. pneumophila treated with KKL-35 for 24 h and of the *trans*-translation-deficient *ssrA*^ind^ mutant deprived of IPTG for 24 h. (B) Activity of KKL-35 against L. pneumophila strains deficient for *trans*-translation. Representative growth curves of the wild-type strain, the *ssrA*^ind^ mutant in the presence or absence of IPTG, and the *ssrA*^ind^ mutant rescued by expression of E. coli ArfA are shown. MICs were determined three times independently on the basis of the absorbance reading.

A lack of *trans*-translation increases the sensitivity to ribosome-targeting antibiotics in E. coli (38, 39) and in L. pneumophila (32). The L. pneumophila *ssrA*^ind^ mutant strain, carrying an IPTG (isopropyl-β-d-thiogalactopyranoside)-inducible allele of the tmRNA-encoding gene *ssrA*, is unable to grow if IPTG is not supplied in the medium (32). Low levels of IPTG allow growth with artificially reduced levels of tmRNA, resulting in increased susceptibility to erythromycin and chloramphenicol (32). A complete lack of *trans*-translation may further increase the sensitivity of L. pneumophila to these antibiotics. Thus, we anticipated that KKL-35 could be synergistic with erythromycin and chloramphenicol. To determine a potential synergy, we performed a checkerboard analysis (40). Interestingly, the MICs of erythromycin (0.125 mg/liter) and chloramphenicol (1 mg/liter) were not affected by KKL-35, indicating the absence of synergy (fractional inhibitory concentration index [FICI] = 2). Thus, unlike the genetic alteration of *trans*-translation, KKL-35 does not potentiate the activity of ribosome-targeting antibiotics. Another phenotype of L. pneumophila cells genetically deprived of tmRNA is extended filamentation, indicating that *trans*-translation is required for cell division (32). In contrast to L. pneumophila cells defective for *trans*-translation, L. pneumophila cells treated with KKL-35 at concentrations at, below, or above the MIC still displayed a normal morphology (Fig. 3A). The inability of KKL-35 to reproduce the phenotypes associated with a loss of *trans*-translation suggests that its potent antibiotic activity is not primarily linked to inhibition of *trans*-translation.

### KKL-35 is equally active against L. pneumophila lacking a *trans*-translation ability.

To test whether the antibiotic activity of KKL-35 was linked to the inhibition of *trans*-translation, we tested the activity of KKL-35 against the L. pneumophila *ssrA*^ind^ mutant strain. When IPTG was supplied at high concentrations, tmRNA was expressed at nearly normal levels, and the strain grew like the wild-type strain. As expected, in the presence of IPTG this strain was as sensitive to KKL-35 as the wild-type strain (MIC = 0.04 mg/liter) (Fig. 3B). In the absence of IPTG, the growth of this strain was strongly impaired, yet despite its low levels of tmRNA, the strain was not more sensitive to KKL-35. Ectopic expression of the alternate ribosome-rescue system ArfA from E. coli could restore the growth of the *ssrA*^ind^ strain in the absence of IPTG. Under these conditions, the strain does not produce tmRNA and is therefore deficient in *trans*-translation (32). Even though it did not require *trans*-translation for growth, the MIC of KKL-35 for this strain remained identical to that for the wild-type strain (Fig. 3B).

### L. pneumophila does not acquire resistance to KKL-35.

**FIG 4:**
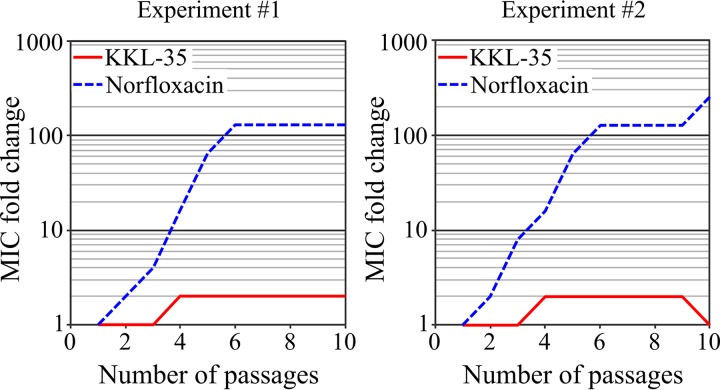
L. pneumophila does not acquire resistance to KKL-35. In two different experiments (several weeks apart), two different lineages were founded from L. pneumophila strain Paris and propagated by serial passages in the presence of KKL-35 or norfloxacin. The MIC was determined at each passage and is presented relative to the initial MIC (norfloxacin, 0.25 mg/liter; KKL-35, 0.04 mg/liter).

*In vitro* selection of resistance is a common way to identify and characterize potential resistance determinants. Plating of a large number of bacteria on solid medium containing an antibiotic at concentrations above the MIC (e.g., rifampin, streptomycin) often allows the isolation of resistant mutants when resistance is conferred by a single mutation. Consistent with published data on E. coli (33), this strategy failed to produce mutants resistant to KKL-35 even when up to 10^10^ L. pneumophila cells were plated on GYE plates containing KKL-35 at concentrations of 2 to 8 times the MIC (0.08 to 032 g/liter). Occasionally, colonies could be obtained on GYE plates with KKL-35 at the MIC (0.04 g/liter), but the colonies could not grow again on freshly prepared GYE plates with the same concentration of KKL-35 (data not shown). These colonies likely emerged because KKL-35 degrades over time. Continuous culture of a bacterial population in the presence of increasing concentrations of antibiotics represents an alternate approach when several mutations are required to confer resistance. In L. pneumophila, this method has been used to characterize the mutational path to resistance to fluoroquinolones and macrolides (7, 8). In agreement with previous reports, in two independent experiments, we observed here a 500-fold increase in the MIC of norfloxacin in only six passages (about 30 generations) (Fig. 4). In contrast, no significant increase in the MIC of KKL-35 was obtained even after 10 passages (over 60 generations) (Fig. 4). Thus, in the experimental setup tested, L. pneumophila could not acquire resistance to KKL-35.

## DISCUSSION

We found KKL-35 to display potent antibiotic activity against L. pneumophila with a MIC of 0.04 mg/liter (0.125 μM). KKL-35 showed significant bactericidal activity and was found to retain normal activity against the different strains of erythromycin-resistant L. pneumophila tested. In addition, and in contrast to the findings for fluoroquinolones and macrolides, L. pneumophila did not develop resistance *in vitro*. Supporting its potential use for the treatment of LD, KKL-35 could stop L. pneumophila from multiplying within monocyte-derived human macrophages. This indicates that KKL-35 is able to cross the biological membranes of the macrophage to reach intracellular L. pneumophila.

KKL-35, along with other oxadiazoles (KKL-10 and KKL-40), was identified in a high-throughput screen for inhibitors of *trans*-translation activity *in vitro* and was initially found to display antibiotic activity against several species. Oxadiazoles have since shown potent antibiotic activity against additional pathogens, such as Francisella tularensis, Bacillus anthracis, or Mycobacterium tuberculosis (34, 41, 42). The antibiotic activity of oxadiazoles against these pathogens was assumed to derive from inhibition of *trans*-translation but was not demonstrated. For L. pneumophila, several results led us to question the link between the inhibition of *trans*-translation and antibiotic activity. First, no synergy between KKL-35 and ribosome-targeting antibiotics was found, whereas we previously found that a reduction in tmRNA levels led to an increased susceptibility of L. pneumophila to such antibiotics (32). Second, L. pneumophila cells treated with KKL-35 did not display the filamentation phenotype observed in cells lacking tmRNA. Most importantly, the MICs were identical when KKL-35 was tested with a wild-type strain, with a growth-affected mutant expressing low levels of tmRNA, or with a mutant that does not require *trans*-translation because tmRNA was replaced by an alternative ribosome-rescue system (ArfA from E. coli). Taken together, our data indicate that *trans*-translation is not the primary target of KKL-35 in L. pneumophila and probably also in other bacteria. Indeed, similar results have been obtained with E. coli, in which KKL-35 is equally effective against strains deficient for *trans*-translation (43). A novel double-fluorescent reporter system for the simultaneous and specific detection of *trans*-translation and proteolysis activities showed that KKL-35 has no direct effect on *trans*-translation (43). Altogether, the data challenge the initial report that KKL-35 is a *trans*-translation inhibitor and that *trans*-translation is a promising antibiotic target (33). The molecular target of KKL-35 has not yet been identified, but the inability to obtain resistant mutants indicates that this target is not prone to support viable mutations. While this is a valuable property for an antibacterial agent, this hampered our efforts to identify the true target of KKL-35 in L. pneumophila. In conclusion, KKL-35 is an effective, broad-spectrum antibacterial agent active against the intracellular pathogen L. pneumophila. However, further studies are needed to better understand its mechanism of action and to assess further the potential of oxadiazoles in treatment.

## MATERIALS AND METHODS

### Strains, growth media, and antibiotics used.

The strains used in this study included L. pneumophila clinical isolates Paris (CIP 107629), Lens (CIP 108286), Philadelphia-1, Lorraine (CIP 108729), and 130b, as well as L. longbeachae ATCC 33484, L. dumoffii ATCC 35280, and L. micdadei ATCC 33218. L. pneumophila strain Paris, which is resistant to erythromycin and azithromycin, was obtained from a previous work (8). L. pneumophila Paris was transformed with the plasmid pX5, a pMMB207C derivative harboring the *gfp^+^* gene under the control of a strong constitutive promoter, and was used for live monitoring of intracellular multiplication by reading of the fluorescence. The *ssrA*^ind^ and *ssrA*^ind^/pArfA tmRNA mutant strains were previously described (32). ACES [*N*-(2-acetamido)-2-aminoethanesulfonic acid]-yeast extract (AYE) broth medium was prepared with 10 g/liter ACES, 12 g/liter yeast extract, 0.3 g/liter iron(III) pyrophosphate, and 0.5 g/liter l-cysteine. The pH was adjusted to 6.9 with KOH, and the solution was filter sterilized and kept away from light and at 4°C. ACES-buffered charcoal-yeast extract (CYE) plates were prepared by combining a 2-fold concentrate of ACES and yeast extract (20 g/liter each in the concentrate) with the same volume of an autoclaved solution of 30 g/liter agar and 4 g/liter charcoal (final concentrations, 15 g/liter agar and 2 g/liter charcoal). The medium was then complemented with 0.25 g/liter filtered iron(III) nitrate and 0.4 g/liter l-cysteine, and the mixture was poured to produce CYE plates. Alternatively, the agar-charcoal solution was replaced by an autoclaved guar gum solution in distilled water (2× concentrate at 10 g/liter; final concentration, 5 g/liter) to produced guar gum-yeast extract (GYE) plates. Unless indicated otherwise, cultures on CYE (or GYE) were incubated for 72 h at 37°C in air and then patched onto CYE again for 24 h to obtain fresh cultures before the experiments were performed. When appropriate, chloramphenicol (5 μg/ml) was added to the medium. A stock solution of KKL-35 (Ambinter, Orléans, France) was prepared at 10 mM (3.2 g/liter) in dimethyl sulfoxide (DMSO) and stored at −20°C. The highest concentration of KKL-35 used was 10 mg/liter (Fig. 2A) with U937 cells or 1.5 mg/liter with L. pneumophila, corresponding to final concentrations of DMSO of 0.3% and 0.05%, respectively.

### Time-kill assay and determination of MICs.

For the time-kill assay, L. pneumophila strain Paris was resuspended in AYE medium at 3 × 10^6^ CFU/ml with a range of 2-fold dilutions of KKL-35. The tubes were then incubated at 37°C in air with shaking. Every 24 h, serial dilutions were plated on CYE agar and the numbers of CFU were counted. For MIC determination, no CLSI guidelines are available for testing the antibiotic susceptibility of Legionella strains. EUCAST guidelines were recently published but are based on the gradient strip test. KKL-35 strip tests are not commercially available, and we found the charcoal of CYE medium to seriously impede KKL-35 activity. Therefore, we used the previously described AYE broth microdilution method for MIC determination (44). Briefly, strains were resuspended in AYE medium and placed into the wells of a 96-well polystyrene plate, and a range of 2-fold dilutions of KKL-35 was added to the cultures. The inoculum (10^6^ CFU/ml) was verified by plating and counting of serial dilutions of the cultures at the beginning of the experiment. The 96-well plate was sealed with a Breathe-Easy membrane (Sigma-Aldrich) to prevent evaporation and was incubated for 48 h at 37°C in air with no agitation. At 48 h, the MICs were determined visually as the lowest concentrations inhibiting bacterial growth.

### Evaluation of synergistic activity.

The checkerboard broth microdilution method was used to evaluate possible synergistic activity between KKL-35 and chloramphenicol or erythromycin against L. pneumophila strain Paris. The bacteria were inoculated in AYE medium in a 96-well polystyrene plate containing a 2-fold range of concentrations of KKL-35 in columns crossing a range of concentrations of another antibiotic in rows. The plate was then incubated for 48 h in a Tecan Infinite M200Pro reader at 37°C with both agitation and reading of the absorbance at 600 nm every 10 min. The growth value was defined as the highest absorbance reading recorded during growth kinetics. Compared to the classic qualitative evaluation of growth by visual observation, this method allowed a quantitative measure of growth to be obtained. Growth inhibition was defined as a maximal absorbance value that was <10% of the value for the positive control. The fractional inhibitory concentration index (FICI) was interpreted in the following way: an FICI of ≤0.5 indicated synergy, an FICI of >4.0 indicated antagonism, and an FICI of >0.5 to 4 indicated no interaction (40).

### Activity of KKL-35 on intracellular growth.

U937 cells grown in RPMI 1640 containing 10% fetal calf serum (FCS) were differentiated into human macrophages by addition of phorbol 12-myristate 13-acetate (PMA) at 100 ng/ml and then seeded into 96-well polystyrene plates for 3 days (10^6^ cells/well). At 4 h before infection, the medium was replaced with fresh medium with 10% FCS. L. pneumophila strain Paris was plated from a glycerol stock at −80°C onto CYE plates, incubated at 37°C in air for 72 h, and then plated again onto CYE plates for 24 h to obtain a fresh culture. At 4 h before infection, the bacteria were resuspended in RPMI 1640 and incubated at 37°C. Infection of macrophages was performed by replacing their medium with RPMI 1640 and 2% FCS containing L. pneumophila at a multiplicity of infection of 10. The plates were centrifuged for 10 min at 1,000 × *g* and then incubated at 37°C with 5% CO_2_ for 72 h. Micrographs were taken with an inverted microscope (Nikon Eclipse TS100). Live monitoring of infection of U937 macrophages was performed as described above, except that GFP-producing L. pneumophila strain Paris/pX5 was used and the infection was performed in CO_2_-independent medium after differentiation and was monitored by use of a Tecan Infinite M200Pro plate reader. The plate was incubated in the reader at 37°C, and GFP fluorescence levels were automatically monitored every hour for 72 h at an excitation wavelength of 470 nm and an emission wavelength of 520 nm.

### Selection of resistant mutants by serial passages.

Two different lineages were founded from L. pneumophila strain Paris and propagated by serial passages in the presence of KKL-35 or norfloxacin, as previously described (7, 8). Briefly, a suspension of L. pneumophila strain Paris in AYE was added to a concentration of 10^8^ CFU/ml in a 24-well polystyrene plate with 2-fold KKL-35 or norfloxacin concentrations ranging from 0.5 time to 8 times the MIC that was determined for the parental strain (norfloxacin, 0.25 mg/liter; KKL-35, 0.04 mg/liter). The plates were sealed with a Breathe-Easy membrane (Sigma-Aldrich) and incubated for 4 days at 37°C in air without agitation, after which the MIC was noted for each antibiotic. A 1:40 dilution of the bacteria from the well with the highest antibiotic concentration in which growth was observable was transferred to a new plate containing 2-fold KKL-35 or norfloxacin concentrations ranging from 0.5 time to 8 times the MIC of the previous cycle. Serial passages were repeated 10 times, and the experiment was performed twice independently.

## References

[1] European Centre for Disease Prevention and Control. 2016 Legionnaires' disease in Europe, 2014. European Centre for Disease Prevention and Control, Stockholm, Sweden.

[2] Woodhead M, Blasi F, Ewig S, Garau J, Huchon G, Ieven M, Ortqvist A, Schaberg T, Torres A, van der Heijden G, Read R, Verheij TJM, Joint Taskforce of the European Respiratory Society and European Society for Clinical Microbiology and Infectious Diseases. 2011 Guidelines for the management of adult lower respiratory tract infections—full version. Clin Microbiol Infect 17(Suppl 6):E1–E59. doi:10.1111/j.1469-0691.2011.03672.x. PMC7128977 21951385

[3] Mandell LA, Wunderink RG, Anzueto A, Bartlett JG, Campbell GD, Dean NC, Dowell SF, File TM, Musher DM, Niederman MS, Torres A, Whitney CG, Infectious Diseases Society of America, American Thoracic Society. 2007 Infectious Diseases Society of America/American Thoracic Society consensus guidelines on the management of community-acquired pneumonia in adults. Clin Infect Dis 44(Suppl 2):S27–S72. doi:10.1086/511159. 17278083 PMC7107997

[4] Phin N, Parry-Ford F, Harrison T, Stagg HR, Zhang N, Kumar K, Lortholary O, Zumla A, Abubakar I 2014 Epidemiology and clinical management of Legionnaires' disease. Lancet Infect Dis 14:1011–1021. doi:10.1016/S1473-3099(14)70713-3. 24970283

[5] Onody C, Matsiota-Bernard P, Nauciel C 1997 Lack of resistance to erythromycin, rifampicin and ciprofloxacin in 98 clinical isolates of Legionella pneumophila. J Antimicrob Chemother 39:815–816. doi:10.1093/jac/39.6.815. 9222053

[6] Jonas D, Engels I, Hartung D, Beyersmann J, Frank U, Daschner FD 2003 Development and mechanism of fluoroquinolone resistance in Legionella pneumophila. J Antimicrob Chemother 51:275–280. doi:10.1093/jac/dkg054. 12562691

[7] Almahmoud I, Kay E, Schneider D, Maurin M 2009 Mutational paths towards increased fluoroquinolone resistance in Legionella pneumophila. J Antimicrob Chemother 64:284–293. doi:10.1093/jac/dkp173. 19474069

[8] Descours G, Ginevra C, Jacotin N, Forey F, Chastang J, Kay E, Etienne J, Lina G, Doublet P, Jarraud S 2017 Ribosomal mutations conferring macrolide resistance in Legionella pneumophila. Antimicrob Agents Chemother 61:e02188-16. doi:10.1128/AAC.02188-16. 28069647 PMC5328525

[9] Shadoud L, Almahmoud I, Jarraud S, Etienne J, Larrat S, Schwebel C, Timsit J-F, Schneider D, Maurin M 2015 Hidden selection of bacterial resistance to fluoroquinolones in vivo: the case of Legionella pneumophila and humans. EBioMedicine 2:1179–1185. doi:10.1016/j.ebiom.2015.07.018. 26501115 PMC4588375

[10] Bruin JP, Koshkolda T, IJzerman EPF, Lück C, Diederen BMW, Den Boer JW, Mouton JW 2014 Isolation of ciprofloxacin-resistant Legionella pneumophila in a patient with severe pneumonia. J Antimicrob Chemother 69:2869–2871. doi:10.1093/jac/dku196. 24898020

[11] Keiler KC, Alumasa JN 2013 The potential of *trans*-translation inhibitors as antibiotics. Future Microbiol 8:1235–1237. doi:10.2217/fmb.13.110. 24059913

[12] Keiler KC 2015 Mechanisms of ribosome rescue in bacteria. Nat Rev Microbiol 13:285–297. doi:10.1038/nrmicro3438. 25874843

[13] Himeno H, Nameki N, Kurita D, Muto A, Abo T 2015 Ribosome rescue systems in bacteria. Biochimie 114:102–112. doi:10.1016/j.biochi.2014.11.014. 25446863

[14] Giudice E, Gillet R 2013 The task force that rescues stalled ribosomes in bacteria. Trends Biochem Sci 38:403–411. doi:10.1016/j.tibs.2013.06.002. 23820510

[15] Feaga HA, Viollier PH, Keiler KC 2014 Release of nonstop ribosomes is essential. mBio 5:e01916-14. doi:10.1128/mBio.01916-14. 25389176 PMC4235212

[16] Keiler KC, Feaga HA 2014 Resolving nonstop translation complexes is a matter of life or death. J Bacteriol 196:2123–2130. doi:10.1128/JB.01490-14. 24706739 PMC4054194

[17] Hudson CM, Lau BY, Williams KP 2014 Ends of the line for tmRNA-SmpB. Front Microbiol 5:421. doi:10.3389/fmicb.2014.00421. 25165464 PMC4131195

[18] Felden B, Himeno H, Muto A, McCutcheon JP, Atkins JF, Gesteland RF 1997 Probing the structure of the Escherichia coli 10Sa RNA (tmRNA). RNA 3:89–103. 8990402 PMC1369465

[19] Keiler KC, Waller PRH, Sauer RT 1996 Role of a peptide tagging system in degradation of proteins synthesized from damaged messenger RNA. Science 271:990–993. doi:10.1126/science.271.5251.990. 8584937

[20] Karzai AW, Susskind MM, Sauer RT 1999 SmpB, a unique RNA-binding protein essential for the peptide-tagging activity of SsrA (tmRNA). EMBO J 18:3793–3799. doi:10.1093/emboj/18.13.3793. 10393194 PMC1171456

[21] Sundermeier TR, Dulebohn DP, Cho HJ, Karzai AW 2005 A previously uncharacterized role for small protein B (SmpB) in transfer messenger RNA-mediated *trans*-translation. Proc Natl Acad Sci U S A 102:2316–2321. doi:10.1073/pnas.0409694102. 15699355 PMC549014

[22] Felden B, Gillet R 2011 SmpB as the handyman of tmRNA during *trans*-translation. RNA Biol 8:440–449. doi:10.4161/rna.8.3.15387. 21558793

[23] Herman C, Thévenet D, Bouloc P, Walker GC, D'Ari R 1998 Degradation of carboxy-terminal-tagged cytoplasmic proteins by the Escherichia coli protease HflB (FtsH). Genes Dev 12:1348–1355. doi:10.1101/gad.12.9.1348. 9573051 PMC316767

[24] Gottesman S, Roche E, Zhou Y, Sauer RT 1998 The ClpXP and ClpAP proteases degrade proteins with carboxy-terminal peptide tails added by the SsrA-tagging system. Genes Dev 12:1338–1347. doi:10.1101/gad.12.9.1338. 9573050 PMC316764

[25] Dougan DA, Weber-Ban E, Bukau B 2003 Targeted delivery of an ssrA-tagged substrate by the adaptor protein SspB to its cognate AAA+ protein ClpX. Mol Cell 12:373–380. doi:10.1016/j.molcel.2003.08.012. 14536077

[26] Karzai AW, Sauer RT 2001 Protein factors associated with the SsrA · SmpB tagging and ribosome rescue complex. Proc Natl Acad Sci U S A 98:3040–3044. doi:10.1073/pnas.051628298. 11248028 PMC30603

[27] Richards J, Mehta P, Karzai AW 2006 RNase R degrades non-stop mRNAs selectively in an SmpB-tmRNA-dependent manner. Mol Microbiol 62:1700–1712. doi:10.1111/j.1365-2958.2006.05472.x. 17087776

[28] Keiler KC 2008 Biology of *trans*-translation. Annu Rev Microbiol 62:133–151. doi:10.1146/annurev.micro.62.081307.162948. 18557701

[29] Chadani Y, Ono K, Kutsukake K, Abo T 2011 Escherichia coli YaeJ protein mediates a novel ribosome-rescue pathway distinct from SsrA- and ArfA-mediated pathways. Mol Microbiol 80:772–785. doi:10.1111/j.1365-2958.2011.07607.x. 21418110

[30] Chadani Y, Ono K, Ozawa S-I, Takahashi Y, Takai K, Nanamiya H, Tozawa Y, Kutsukake K, Abo T 2010 Ribosome rescue by Escherichia coli ArfA (YhdL) in the absence of *trans*-translation system. Mol Microbiol 78:796–808. doi:10.1111/j.1365-2958.2010.07375.x. 21062370

[31] Handa Y, Inaho N, Nameki N 2011 YaeJ is a novel ribosome-associated protein in Escherichia coli that can hydrolyze peptidyl-tRNA on stalled ribosomes. Nucleic Acids Res 39:1739–1748. doi:10.1093/nar/gkq1097. 21051357 PMC3061065

[32] Brunel R, Charpentier X 2016 *trans*-Translation is essential in the human pathogen Legionella pneumophila. Sci Rep 6:37935. doi:10.1038/srep37935. 27892503 PMC5124942

[33] Ramadoss NS, Alumasa JN, Cheng L, Wang Y, Li S, Chambers BS, Chang H, Chatterjee AK, Brinker A, Engels IH, Keiler KC 2013 Small molecule inhibitors of *trans*-translation have broad-spectrum antibiotic activity. Proc Natl Acad Sci U S A 110:10282–10287. doi:10.1073/pnas.1302816110. 23733947 PMC3690859

[34] Goralski TDP, Dewan KK, Alumasa JN, Avanzato V, Place DE, Markley RL, Katkere B, Rabadi SM, Bakshi CS, Keiler KC, Kirimanjeswara GS 2016 Inhibitors of ribosome rescue arrest growth of Francisella tularensis at all stages of intracellular replication. Antimicrob Agents Chemother 60:3276–3282. doi:10.1128/AAC.03089-15. 26953190 PMC4879415

[35] Kim E-H, Charpentier X, Torres-Urquidy O, McEvoy MM, Rensing C 2009 The metal efflux island of Legionella pneumophila is not required for survival in macrophages and amoebas. FEMS Microbiol Lett 301:164–170. doi:10.1111/j.1574-6968.2009.01813.x. 19895645

[36] Roy CR, Berger KH, Isberg RR 1998 Legionella pneumophila DotA protein is required for early phagosome trafficking decisions that occur within minutes of bacterial uptake. Mol Microbiol 28:663–674. doi:10.1046/j.1365-2958.1998.00841.x. 9632267

[37] Wiater LA, Dunn K, Maxfield FR, Shuman HA 1998 Early events in phagosome establishment are required for intracellular survival of Legionella pneumophila. Infect Immun 66:4450–4460. 9712800 10.1128/iai.66.9.4450-4460.1998 PMC108538

[38] Luidalepp H, Hallier M, Felden B, Tenson T 2005 tmRNA decreases the bactericidal activity of aminoglycosides and the susceptibility to inhibitors of cell wall synthesis. RNA Biol 2:70–74. doi:10.4161/rna.2.2.2020. 17132943

[39] Li J, Ji L, Shi W, Xie J, Zhang Y 2013 *trans*-Translation mediates tolerance to multiple antibiotics and stresses in Escherichia coli. J Antimicrob Chemother 68:2477–2481. doi:10.1093/jac/dkt231. 23812681 PMC3797643

[40] Odds FC 2003 Synergy, antagonism, and what the chequerboard puts between them. J Antimicrob Chemother 52:1. doi:10.1093/jac/dkg301. 12805255

[41] Alumasa JN, Goralski TDP, Keiler KC 2017 Tetrazole-based *trans*-translation inhibitors kill Bacillus anthracis spores to protect host cells. Antimicrob Agents Chemother 61:e01199-17. doi:10.1128/AAC.01199-17. 28760903 PMC5610534

[42] Alumasa JN, Manzanillo PS, Peterson ND, Lundrigan T, Baughn AD, Cox JS, Keiler KC 2017 Ribosome rescue inhibitors kill actively growing and nonreplicating persister Mycobacterium tuberculosis cells. ACS Infect Dis 3:634–644. doi:10.1021/acsinfecdis.7b00028. 28762275 PMC5594445

[43] Macé K, Demay F, Guyomar C, Georgeault S, Giudice E, Goude R, Trautwetter A, Ermel G, Blanco C, Gillet R 2017 A genetic tool to quantify *trans*-translation activity in vivo. J Mol Biol 429:3617–3625. doi:10.1016/j.jmb.2017.10.007. 29031699

[44] Baltch AL, Smith RP, Ritz W 1995 Inhibitory and bactericidal activities of levofloxacin, ofloxacin, erythromycin, and rifampin used singly and in combination against Legionella pneumophila. Antimicrob Agents Chemother 39:1661–1666. doi:10.1128/AAC.39.8.1661. 7486896 PMC162803

